# Reconstruction of ancestral protein sequences and its applications

**DOI:** 10.1186/1471-2148-4-33

**Published:** 2004-09-17

**Authors:** Wei Cai, Jimin Pei, Nick V Grishin

**Affiliations:** 1 Howard Hughes Medical Institute, University of Texas Southwestern Medical Center at Dallas, 5323 Harry Hines Blvd., Dallas, TX. 75390-9050, USA; 2 Department of Biochemistry, University of Texas Southwestern Medical Center at Dallas, 5323 Harry Hines Blvd., Dallas, TX. 75390-9050, USA

## Abstract

**Background:**

Modern-day proteins were selected during long evolutionary history as descendants of ancient life forms. *In silico *reconstruction of such ancestral protein sequences facilitates our understanding of evolutionary processes, protein classification and biological function. Additionally, reconstructed ancestral protein sequences could serve to fill in sequence space thus aiding remote homology inference.

**Results:**

We developed ANCESCON, a package for distance-based phylogenetic inference and reconstruction of ancestral protein sequences that takes into account the observed variation of evolutionary rates between positions that more precisely describes the evolution of protein families. To improve the accuracy of evolutionary distance estimation and ancestral sequence reconstruction, two approaches are proposed to estimate position-specific evolutionary rates. Comparisons show that at large evolutionary distances our method gives more accurate ancestral sequence reconstruction than PAML, PHYLIP and PAUP*. We apply the reconstructed ancestral sequences to homology inference and functional site prediction. We show that the usage of hypothetical ancestors together with the present day sequences improves profile-based sequence similarity searches; and that ancestral sequence reconstruction methods can be used to predict positions with functional specificity.

**Conclusions:**

As a computational tool to reconstruct ancestral protein sequences from a given multiple sequence alignment, ANCESCON shows high accuracy in tests and helps detection of remote homologs and prediction of functional sites. ANCESCON is freely available for non-commercial use. Pre-compiled versions for several platforms can be downloaded from .

## Background

Present-day protein sequences can be used to reconstruct ancestral sequences based on a model of sequence evolution. Such knowledge about ancestral sequences is helpful for understanding the evolutionary processes as well as the functional aspects of a protein family. Existing methods of ancestral sequence reconstruction can be divided into two main categories: Maximum Parsimony (MP) methods [1,2] and Maximum Likelihood (ML) methods [3-5]. MP methods do not take into account biased substitution patterns between amino acids or different tree branch lengths, and cannot distinguish those equally parsimonious reconstructions [3]. ML methods do not have these limitations and generally give more reliable results than the MP methods [6]. Yang et al. [3] first developed a ML method for ancestral sequence reconstruction. Yang [7] also made a distinction between "joint" reconstruction and "marginal" reconstruction. Joint reconstruction methods intend to find the most likely set of amino acids for all internal nodes at a site, which yields the maximum joint likelihood of the tree [5]. Marginal reconstruction compares the probabilities of different amino acids at an internal node at a site and selects the amino acid that yields the maximum likelihood for the tree at that site. Marginal reconstruction can also compute probabilities of all other amino acids for that node [4]. Koshi and Goldstein [4] developed a fast dynamic programming algorithm for marginal reconstruction in the framework of Bayesian statistics, while Pupko et al. [5] proposed a fast algorithm for joint reconstruction. The computational complexities for both algorithms scale linearly with the number of sequences. Both marginal and joint reconstruction algorithms are implemented in our program.

All reconstruction methods require a phylogenetic tree inferred from a given alignment. The quality of the tree is crucial for the reliability of reconstruction. A number of methods exist for phylogenetic inference, such as maximum likelihood [8], distance-based [9] and parsimony [1]. Distance-based methods have the advantage of being simple and are able to handle a large set of sequences. They require evolutionary distances estimated for all the sequence pairs. The most common method to infer phylogeny from distances is based on the neighbor-joining algorithm [9]. Bruno et al. [10] introduced a distance-based phylogeny reconstruction method called "Weighbor", i.e. "weighted neighbor joining", which takes into account the fact that errors in distance estimates are larger for longer distances. Giving similar results, Weighbor is much faster than ML phylogeny reconstruction. It is also better than other methods such as BIONJ [11] and parsimony [1], in aspects of "long branches attract" and "long branch distracts" problems [10]. Weighbor is used in our program for phylogenetic inference.

Overwhelming evidence exists for substitution rate variation across sites [12-15]. For a protein family, rate heterogeneity reflects the selective pressure imposed by folding, stability and function. Gamma distribution is widely used to model the rate variation among sites [13,16,17] because of its simplicity. Nielsen [18] suggested a method for site-by-site estimation of rate factors by a Maximum Likelihood approach. Rate variation among sites has not been taken into account in the early work of ML reconstruction of ancestral sequences [4,5]. Recently, Pupko et al. [19] introduced rate variation into joint reconstruction by a branch-and-bound algorithm, assuming a gamma distribution of rates among sites. In our package, two methods are proposed to estimate a rate factor for each site. The first one is based on our observation that the substitution rate at a site is correlated with the conservation of the site. The more conserved the site is in a multiple sequence alignment, the smaller its substitution rate is. This empirical method, the result of which we call Alignment-Based rate factors or *α*_*AB*_, relies only on a multiple sequence alignment and a general model of amino acid exchange. The other one is a maximum likelihood method (*α*_*ML*_), which requires a tree. In our implementation, we incorporate *α*_*AB *_or *α*_*ML *_in the joint and marginal reconstruction algorithms [4,5]. *α*_*AB *_is also used in the Maximum Likelihood estimation of evolutionary distances [20] for tree inference.

We implement a method of evolutionary simulation that introduces site-specific rate variations in a natural way by imposing structural and functional constraints [21]. We show by simulations that the reconstruction methods can give reasonable results and that the problem of evolutionary distance underestimation [22] is alleviated by considering rate variation across sites.

Background (or equilibrium) amino acid frequencies (*π*) are usually estimated from the target set of sequences or from large databases of protein families. Background amino acid frequencies estimated from a small dataset tend to have bias, while amino acid frequencies from large databases may not be suitable for the specific protein family under analysis. Here, we propose a ML method to optimize the amino acid frequency vector *π*. The optimized *π *vector can give significant improvement over the likelihood of a alignment.

Information obtained from ancestral sequence reconstruction is used for two applications: homology detection and prediction of functional sites. For homology detection, ancestral sequences represent an enlargement of the sequence space around native sequences. We demonstrate that adding reconstructed ancestral sequences to a native alignment improves the detection of homologs in database searches.

A number of methods have been developed to predict functional sites from amino acid sequences [23,24]. One simple way to infer functional sites is by positional conservation of a multiple sequence alignment [25]. Lichtarge et al. [26] proposed a method called evolutionary trace to predict functional sites by analyzing the conservation of sequence subgroups. Functional divergence during the evolutionary process can be reflected in the variation of amino acid usage across different functional subgroups. We propose a new approach that uses information from ancestral sequence reconstruction to identify sites that are well conserved within individual sub-trees but exhibit variability among different sub-trees. By several examples, we show that these sites frequently contribute to the functional specificity of a protein family.

## Results and discussion

We developed a package (ANCESCON) to reconstruct ancestral protein sequences considering rate variation among sites. Rate factors can be estimated either by an empirical method or by a maximum likelihood method. Consideration of rate variation among sites not only improves evolutionary distance estimation, but also gives more accurate ancestral sequence reconstruction. Ancestral sequences are used to improve profile-based sequence similarity searches. We also propose a new approach to predict positions with functional specificity based on the reconstruction of ancestral sequences.

### Observed *α*, Alignment Based Rate Factor *α *(*α*_*AB*_) and Rate Factor *α *estimated by Maximum Likelihood (*α*_*ML*_)

Evolutionary simulations based on a *Z*-score model introduce rate variation across sites in a natural way by incorporating structural and functional constraints specific for a protein family [21]. The simulation procedure is a Monte Carlo simulation of the amino acid substitution process. The fixation of substitutions is dictated by a simple scoring function, which is derived from the template structure and an alignment of its homologs. The number of substitutions occurring at each site can be recorded during the simulation process and the observed *α *at a site equals the number of recorded substitutions at that site divided by the average substitution number for all sites. To reduce sampling variance, an average observed *α *vector is calculated from 100 simulations.

For the alignment consisting of all the leaf node sequences generated by the simulation process, an *α*_*AB *_vector was calculated according to equation (11) (for details see Methods). An average *α*_*AB *_vector was derived from 100 simulations. Correlation coefficient between the average *α*_*AB *_vector and the average observed *α *vector was high (data not shown). However, we found that for large observed *α *values, the corresponding *α*_*AB *_values were smaller. A constant *β *was introduced to correct this underestimation in equation (11).



Here, *α*_*i *_is Alignment-Based rate factor at site *i*. *K *is the number of sites in a given alignment. *C*_*i *_is the value assigned to site *i *(for details see Methods).

**Figure 1:**
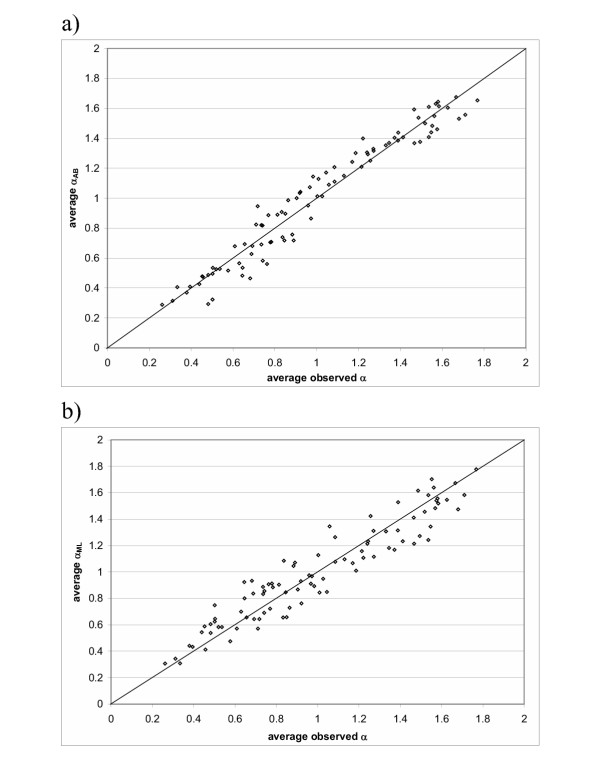
**a) Correlation between average *α*_*AB *_and average observed *α*. b) Correlation between average *α*_*ML *_and average observed *α*. ***α*_*AB *_is Alignment-Based rate factor solely depending on the given alignment. *α*_*ML *_is rate factor estimated by maximum likelihood method, which requires an alignment and evolutionary tree inferred from the alignment. The protein family used here is the PDZ domain.

We optimized the *β *value by fitting the average *α*_*AB *_vector and average observed *α *vector to *y *= *x *line. Alignments for three different protein families (trypsin, carboxypeptidase and pdz domain) gave a good empirical estimation for *β *of about 1.3. The relation between this corrected average *α*_*AB *_vector and average observed *α *vector is shown in Figure 1a for a typical example, the pdz domain (correlation coefficient 0.973).

We also estimated an *α*_*ML *_vector for each alignment generated from the simulation (for details see Methods). The average *α*_*ML *_vector shows good correlation with the average observed *α *vector (Figure 1b) (correlation coefficient 0.945). *α*_*AB *_or *α*_*ML *_can be incorporated in likelihood calculation in marginal or joint reconstruction. Table 1 shows that improvement of logarithm likelihood of the alignment is significant when *α*_*AB *_or *α*_*ML *_is used.

**Table 1:** Difference of logarithm likelihood and CPU time when using different *α *vectors

	*α *= 1.0	*α*_*AB*_			*α*_*ML*_		
			Δ*l*	P*		Δ*l*	P*

Logarithm Likelihood	-5324.56	-5087.72	236.84	<0.0001	-4987.27	337.29	<0.0001
CPU Time (s)^+^	213		213			359		

The alignment tested here is a subset of SH2 family. It includes 44 sequences and each sequence contains 83 amino acids (including gaps).

* The likelihood ratio test (LRT) [58] is used to test whether *α*_*AB *_and *α*_*ML *_are significantly different from *α *= 1.0. The difference in number of free parameters between *α*_*AB*_, *α*_*ML *_and *α *= 1.0 model is 82.

^+ ^CPU times were computed on a Dell PowerEdge 8450 server (CPU 700MHz, RAM 8G).

Rate variation across sites can be modeled by assuming that the rate factors follow a certain type of statistical distribution. Gamma distribution [13,27] and its discrete approximations [28] are frequently used for DNA or protein sequences. Rate variation for a protein family reflects different selective pressure at different sites to maintain structure and function. Fewer substitutions are expected to occur in more conserved sites. This hypothesis has prompted us to estimate rate factors (*α*_*AB*_) based on sequence conservation in an empirical way. The *α*_*AB *_is compared and calibrated using the observed *α *as standards. Our method of estimating *α*_*ML *_is similar to the one proposed by Nielson [18]. One problem with site-by-site rate factor estimation is the small sample size at each site, especially with a small alignment. We have used *α*_*AB *_to eliminate outliers with very large *α*_*ML *_estimates (for details see Methods).

### Site-specific rate factors improve distance estimation

**Figure 2:**
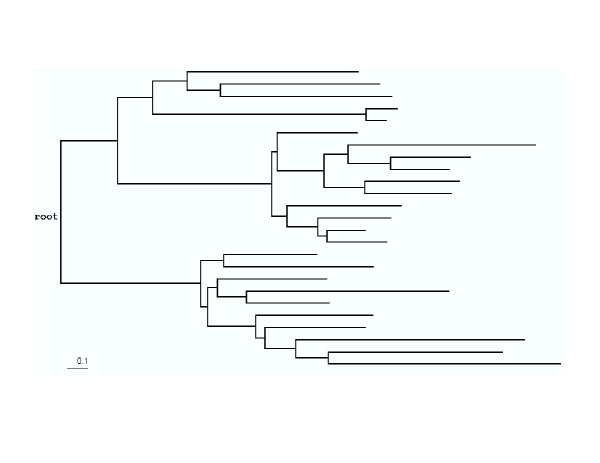
**The tree used to test ancestral sequence reconstruction. **This is an arbitrarily selected evolutionary tree. Evolutionary distances are shown to scale.

**Figure 3:**
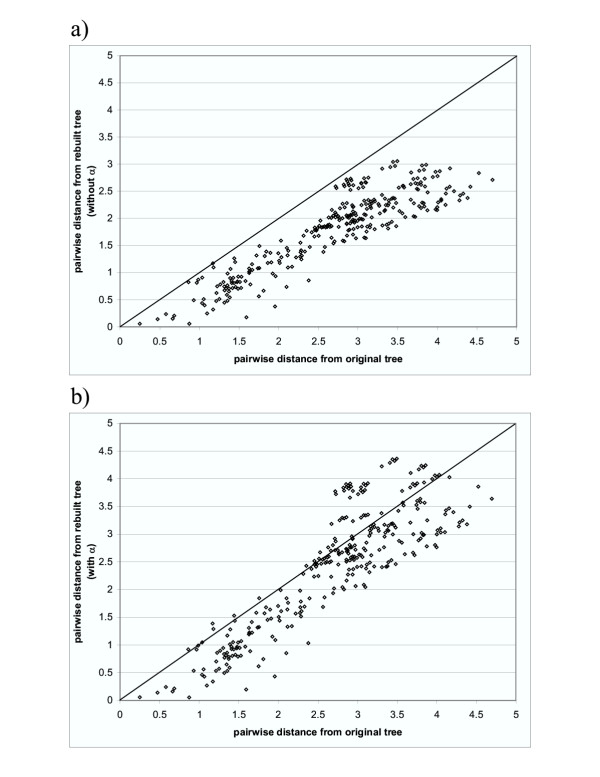
**Comparison of pairwise distances between the rebuilt tree and original tree. a) distance estimation assuming no rate variation among sites; b) distance estimation with *α*_*AB*_. **The rebuilt tree is inferred from the alignment that is generated by evolutionary simulation performed on the original tree. The original tree is arbitrarily selected.

Evolutionary distances tend to be underestimated when rate homogeneity among sites is assumed [22]. This was tested using the simulation with structural and functional constraints. For the arbitrarily selected tree shown in Figure 2, we obtained leaf node sequences in the simulation and estimated an evolutionary distance for each sequence pair by Maximum Likelihood, either incorporating *α*_*AB *_or setting *α *equal to 1.0 (equation (16)). Evolutionary distances were severely underestimated (average underestimation: 0.894) without considering rate variation among sites (Figure 3a). Introducing *α*_*AB *_in the maximum likelihood method gave more accurate distance estimation (Figure 3b), although the distances were still underestimated, especially for small distances (average underestimation: 0.286). We believe that more accurate distances will give more accurate phylogeny reconstruction using "Weighbor" [10]. Since a tree is required to estimate *α*_*ML*_, *α*_*ML *_is not incorporated in estimating evolutionary distance.

### Optimization of equilibrium frequencies

A continuous minimization method by simulated annealing was used to optimize the equilibrium frequency vector *π*, with the objective function being the logarithm likelihood of the alignment. Our *π *vector optimization program was tested on four alignments, which were taken from the SH2 and SH3 superfamilies in Pfam database (version 7.3) [29]. Two alignments from the SH2 superfamily have 44 and 87 sequences respectively and both alignment lengths are 83 amino acids (including gaps). The other two alignments from SH3 superfamily have 39 and 94 sequences respectively and both alignment lengths are 57 amino acids (including gaps). For each alignment, we ran optimization 3 times starting from different random initial points. The optimized *π *vectors did not converge to exactly the same point, but they had a high correlation with each other (always > 0.95) and the difference of logarithm likelihood function values was small (less than 0.1%). The logarithm likelihood of the alignment, using optimized *π *vector, increased slightly, but significantly (Table 2), compared with the logarithm likelihood using the *π *vector calculated from the alignment.

**Table 2:** Difference of logarithm likelihood and CPU time with and without optimization of *π *vector

	*α*_*AB *_& Calculated *π*	*α*_*AB *_& Optimized *π*	Δ*l*	P*
Logarithm Likelihood	-5087.72	-5055.97	31.75	<0.0001
CPU Time (s)^+^	213	14902		

The alignment tested here is the same alignment used in Table 1. Calculated *π *means frequency vector calculated from the alignment.

* The likelihood ratio test (LRT) [58] is used to test whether optimized *π *is significantly different from calculated *π*. The difference in number of free parameters between these two models is 19.

^+^CPU times were computed on a Dell PowerEdge 8450 server (CPU 700MHz, RAM 8G).

Optimization of the *π *vector is time consuming. The running time for reconstruction with or without optimizing *π *vector is 14,902 seconds and 213 seconds for SH2 alignment (44 sequences), respectively, on a Dell PowerEdge 8450 server (CPU 700MHz, RAM 8G) (Table 2). In our program, the default *π *vector is calculated from the alignment while the user has the option to optimize the *π *vector for ancestral sequence reconstruction.

### Testing reconstruction

**Figure 4:**
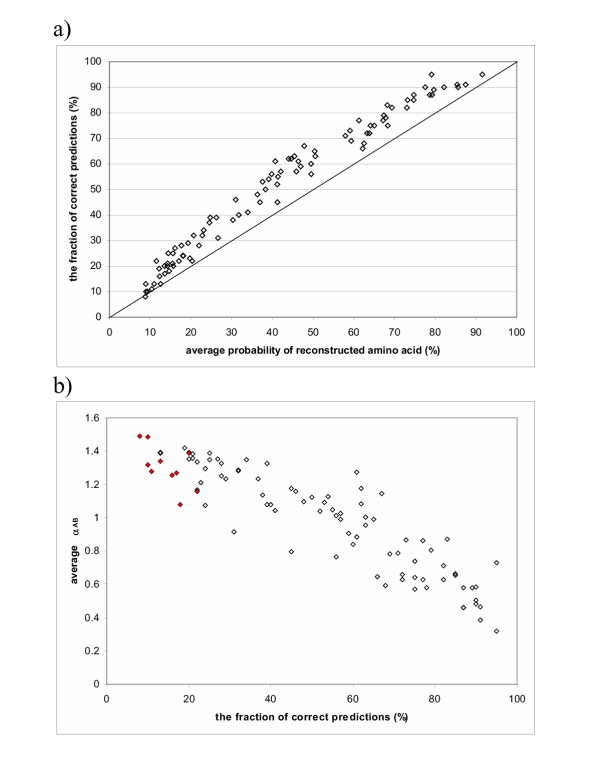
**a) Correlation between the average probability of "the reconstructed amino acid" and the fraction of correct predictions. b) Correlation between the fraction of correct predictions and average *α*_*AB *_at each site. **The protein family used here is the PDZ domain. Red filled points are sites with incorrect reconstruction.

Two different methods for simulations of the evolutionary process were used, as described in Methods, to test the reliability of the reconstruction results. In the first simulation method, starting from a randomly generated root sequence, we simulated the evolutionary process to obtain leaf node sequences based on a tree and a rate matrix. This process was repeated 100 times for a given root sequence *R *to produce 100 alignments consisting of all leaf node sequences. For each of the 100 alignments, we used the marginal reconstruction method to obtain an amino acid probability vector for each site at the root. To reduce sampling variance, the amino acid probability vector was averaged over the 100 simulation trials. At each site, the amino acid with the highest average probability was chosen as our result of the "reconstructed amino acid" at that site. All "reconstructed amino acids" formed the reconstructed sequences *R'*. There is no difference between *R *and *R'*, that is, the accuracy of reconstruction is 100% for the tree shown in Figure 2. For each individual simulation and its reconstruction, we checked the amino acid with the highest probability in the reconstructed probability vector of the root. If it is indeed the "reconstructed amino acid", the prediction for that simulation is correct according to the average reconstructed results. The fraction of individual predictions that are correct according to the average reconstructed results is almost always higher than the average probability of the "reconstructed amino acid", suggesting that the average probability of the "reconstructed amino acid" gives a lower estimation of the reconstruction reliability (Figure 4a).

For the second simulation method, we introduced rate heterogeneity across sites with structural and functional constraints [21]. For the same tree, the accuracy of reconstruction was about 90%. Sites with larger substitution rates are expected to have less reliable reconstructions. Figure 4b shows the relationship between the average *α*_*AB *_and the fraction of individual predictions that are correct according to the "reconstructed amino acid". Sites with incorrect "reconstructed amino acids" all have large *α*_*AB *_values. These values reflect the difficulty of reconstructing sites with large numbers of substitutions. The probabilities of the "reconstructed amino acids" are all small for sites with incorrect reconstructions (less than 0.15), suggesting that the information content of the reconstruction is low.

The second simulation method was also used to test ANCESCON along with the reconstruction programs from PAML [30], PHYLIP [31] and PAUP* [32]. All tree topologies used in reconstruction tests were inferred from real alignments. All original root sequences were taken from PDB database [33]. We had three different types of alignment testing sets. The first testing set used the same tree topology but different root sequences to generate 100 alignments (for details see Methods). The second testing set used the same root sequence but different tree topologies. The third testing set randomly selected a root sequence and a tree topology to generate 100 alignments. After 100 alignments were generated, we reconstructed the root sequence for each alignment and found the consensus root sequence for the 100 reconstructed root sequences. Finally, the consensus root sequence was compared with the original root sequence to calculate the reconstruction accuracy, i.e. the fraction of correctly reconstructed sites for the root sequence. In addition, for the third test, the paired t-test was used to calculate the one-tail probability between ANCESCON and other three methods. In order to make different tree topologies comparable, those trees were scaled to make the average distance from root to all leaf nodes (*d*_*a*_) the same for all trees and equal to the tree of pii1 (a signal transduction protein) (*d*_*a *_= 4.23). If *d*_*a *_was too small (e.g. 0.5), the reconstruction accuracy was always close to 1 for all reconstruction methods used. The value *d*_*a *_= 4.23 was large enough to generate diverse sequences to differentiate 4 different ancestral sequence reconstruction methods.

For ANCESCON we had 3 different parameter settings, which included site-specific rate factors estimated by maximum likelihood method (*α*_*ML*_), Alignment-Based rate factors (*α*_*AB*_) and no rate factors (equal rates among sites). Different parameters were also used for the reconstruction programs from PAML and PHYLIP to find their best reconstructions. For PAML, reconstruction was tested with parameter *α *(rate factor) estimated from alignment and without *α*. For PHYLIP, 4 different parameter settings were tried, which were combinations of with/without *α *estimated from alignment by PAML and with/without branch length dwelling in input tree topology. For PAUP*, default settings were used.

Table 3 shows a comparison of the reconstruction accuracy for these 4 methods. The reconstruction accuracy of ANCESCON with *α*_*ML *_is higher than the other three methods in almost every test. Also the reconstruction accuracy of ANCESCON with *α*_*AB *_and without *α *is comparable with PAML and PHYLIP methods and is much better than PAUP*. For the first testing set, the best average accuracy for ANCESCON is about 0.5, while the best average reconstruction accuracies for PAML, PHYLIP and PAUP* are 0.45, 0.39 and 0.32 respectively. Testing set 2 and 3 produce similar results. Using the paired t-test in the third testing set, we show that ANCESCON method with *α*_*ML *_gives significantly better reconstruction than the other 3 methods. Because the site-specific *α*_*ML *_is very close to the true mutation rate at a site (Figure 1b), using the site-specific *α*_*ML *_can improve our ability to reconstruct the amino acids for ancestral sequences correctly. These reconstruction tests suggest that ANSCESCON may be a better tool to reconstruct ancestral sequences compared to PAML, PHYLIP and PAUP* if the given alignment contains more diverse sequences.

**Table 3:** Ancestral sequence reconstruction accuracy by different programs

Root Seq.	Tree	Leaf Node Num.	Methods									
			
			ANCESCON			PAML		PHYLIP $				PAUP*
				
			*α*_*ML*_	*α*_*AB*_	-*α*	+*α*	-*α*	+L +*α*	-L +*α*	+L -*α*	-L -*α*	
1em2	pii1	25	**0.45**	0.32	0.35	0.41	0.37	0.29	0.27	0.21	0.29	0.26
1g9o	pii1	25	**0.56**	0.46	0.47	0.53	0.53	0.51	0.54	0.40	0.51	0.47
1rgg	pii1	25	0.60	0.42	0.47	0.60	**0.62**	0.47	0.58	0.32	0.56	0.47
1sgt	pii1	25	**0.38**	0.34	0.33	0.33	0.32	0.32	0.33	0.27	0.33	0.32
1zm2	pii1	25	**0.33**	0.29	0.3	0.28	0.25	0.21	0.25	0.21	0.27	0.16
2a8v	pii1	25	**0.62**	0.45	0.42	0.56	0.55	0.44	0.46	0.28	0.50	0.36
2ctb	pii1	25	**0.53**	0.40	0.39	0.41	0.38	0.24	0.24	0.21	0.29	0.22
Average accuracy			**0.496**	0.383	0.390	0.446	0.431	0.354	0.381	0.271	0.393	0.323
2ctb	gef	27	**0.54**	0.37	0.38	0.35	0.35	0.29	0.17	0.24	0.22	0.22
2ctb	LacI	54	**0.66**	0.64	0.57	0.44	0.37	0.49	0.35	0.42	0.33	0.34
2ctb	pdz	39	**0.54**	0.41	0.42	0.44	0.39	0.22	0.34	0.18	0.32	0.22
2ctb	ph	30	**0.79**	0.74	0.75	0.53	0.55	0.45	0.25	0.43	0.37	0.32
2ctb	pii1	25	**0.53**	0.40	0.39	0.41	0.38	0.24	0.24	0.21	0.29	0.22
2ctb	ptb	29	**0.58**	0.39	0.43	0.39	0.38	0.29	0.23	0.26	0.24	0.23
2ctb	sh2	34	**0.61**	0.42	0.40	0.43	0.40	0.30	0.22	0.20	0.27	0.22
2ctb	sh3	43	**0.83**	0.82	0.80	0.62	0.55	0.69	0.45	0.66	0.46	0.54
2ctb	GST	140	**0.76**	0.73	0.73	@	@	#	#	0.47	0.38	0.33
Average accuracy^&^			**0.635**	0.524	0.518	0.451	0.421	0.371	0.281	0.325	0.313	0.289
1em2	pdz	39	**0.45**	0.35	0.36	0.44	0.44	0.29	0.43	0.23	0.4	0.24
1g9o	pii1	25	**0.56**	0.46	0.47	0.53	0.53	0.51	0.54	0.40	0.51	0.47
1rgg	sh2	34	**0.64**	0.48	0.46	0.61	0.61	0.56	0.59	0.34	0.6	0.41
1sgt	gef	27	**0.49**	0.39	0.40	0.48	0.44	0.42	0.44	0.36	0.45	0.41
1zm2	ptb	29	**0.66**	0.47	0.48	0.57	0.57	0.53	0.51	0.32	0.52	0.41
2a8v	ph	30	**0.81**	0.78	**0.81**	0.71	0.74	0.60	0.61	0.50	0.65	0.50
2ctb	LacI	54	**0.66**	0.64	0.57	0.44	0.37	0.49	0.35	0.42	0.33	0.34
Average accuracy			**0.610**	0.510	0.507	0.540	0.529	0.486	0.496	0.367	0.494	0.397
Probability^Δ^				0.0026	0.0023	0.0248	0.0328	0.0007	0.0168	0.0001	0.0143	0.0005

All root sequences are taken from PDB database and the names listed in the table are PDB IDs.

Tree topologies for gef (guanine nucleotide exchange factor), LacI (PurR/LacI family of bacterial transcription factors), pdz, ph, pii1 (a signal transduction protein), ptb, sh2, sh3 and GST (glutathione S-transferase) are inferred from multiple sequence alignments chosen from Pfam database (version 7.3).

All tree topologies are generated from real alignments and the distances are rescaled in order to make the trees comparable.

The value in this table represents the accuracy of reconstruction, i.e. the fraction of correctly reconstructed sites for the root sequence. The best reconstruction accuracy in each test is shown in bold.

*α*_*ML *_means that the site-specific rate factors were estimated by maximum likelihood method.

*α*_*AB *_means that the site-specific rate factors were estimated by our empirical equation based on the given alignment (for details see Methods).

-*α *means that the rate factors were not considered in reconstruction.

+*α *means that the rate factors were considered in reconstruction.

+L means that branch lengths of the input tree were used in reconstruction, while -L means that branch lengths were estimated by the reconstruction program itself.

@: tree topology for GST had 140 leaf nodes that were too many for PAML to run through.

^$^: rate factors estimated by PAML were used by PHYLIP in ancestral sequence reconstruction.

#: tree topology for GST had 140 leaf nodes, which were too many for PAML to estimate rate factors for GST.

^&^:GST is excluded in calculation of the average.

^Δ^: paired t-test method [40] was used to estimate the one-tail probability between ANCESCON and the other three reconstruction methods.

### Ancestral sequences used in homology detection

Thirty-eight OB (Oligonucleotide/oligosaccharide binding)-fold [34] proteins and ten other alignments (adenylyl kinase, gef, globin, pdz, ph, ptb, ras, sh2, sh3 and subtilase) from the Pfam database (version 7.3) [29] were chosen to perform homology detection tests.

Given an alignment with *N *sequences, we had four different methods, "BEST", "SECOND BEST", "SHUFFLE" and "RANDOM", to generate another *N*-1 sequences (for details see Methods). For each combined alignment (2*N*-1 sequences), PSI-BLAST [35] searches were performed starting from each sequence and seeded with the combined alignment (-B option in the program BLASTPGP, e-value cutoff 0.01), and all found hits were pooled together.

The benchmark experiment was PSI-BLAST seeded with the native alignment (*N *sequences). For each type of the four combined alignments, we checked hits not found by the native alignments. New hits were verified to be true positives or false positives by running PSI-BLAST or HMMER [36], followed by manual inspections.

**Figure 5:**
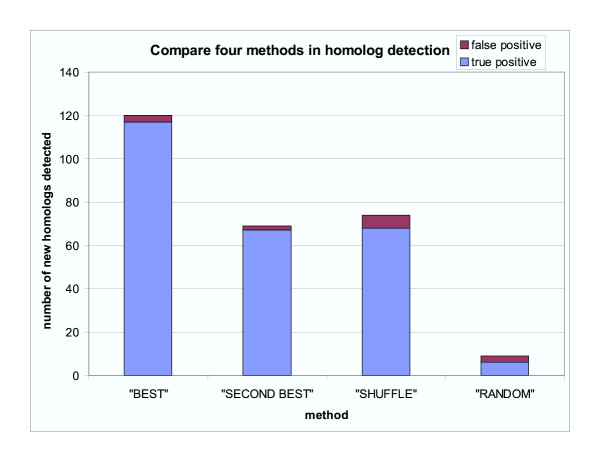
**Comparison of "BEST", "SECOND BEST", "SHUFFLE" and "RANDOM" methods in the number of new homologs detected when compared with the benchmark experiment. **The methods are defined in "Methods" section. The blue portion of the bar shows the number of true positives. The red portion of the bar shows the number of the false positives.

Using the 48 native alignments, a total of 13973 hits were found by the benchmark. Compared to the benchmark, the "BEST" method detected 120 new homologs and the other three methods found 69, 74 and 9 new homologs, respectively (Figure 5). Among those new homologs, "BEST", "SECOND BEST", "SHUFFLE" and "RANDOM" methods had 3, 2, 6 and 3 false positives, respectively (Figure 5). Also, "BEST", "SECOND BEST", "SHUFFLE" and "RANDOM" methods missed 61, 1070, 60 and 7811 homologs as compared to the benchmark.

Adding non-native sequences to the native alignment results in a change of sequence profile for PSI-BLAST searches. Random sequences can dilute the position-specific amino acid exchange characteristics of native alignments. This effect should not improve the profile. Indeed, few new homologs are found by the "RANDOM" method. However, sequences generated by shuffling each position of the native alignment have the same conservation properties as the native alignment, and the "SHUFFLE" method detects a total of 74 new homologs. Two effects may account for this finding. First, addition of shuffled sequences to the native alignment can slightly change the estimates of pseudocount frequencies of amino acids and thus the position specific scoring matrix [35]. Second, the new version of PSI-BLAST program uses composition-based statistics with e-value estimation related to the composition of the query sequence [37]. Each shuffled sequence has its own amino acid composition that is different from the native sequences. This difference can affect the e-values of hits. The "BEST" method detects the most number of new homologs, suggesting that the reconstructed ancestral sequences resemble the native sequences. Ancestral sequences may therefore be more similar to some remote homologs than to the native sequences. The "SECOND BEST" method detects less new homologs than the "BEST" method but more than the "RANDOM" method, suggesting that the second most probable amino acids in reconstruction can still reflect some properties of native sequences. Table 4 shows homology detection results of OB-fold structures using reconstructed ancestral sequences.

**Table 4:** Homology detection results of OB-fold structures using reconstructed ancestral sequences

SCOP Superfamily/family	PDB structure	New homologs	NCBI annotation
Nucleic acid-binding proteins/ Anticodon-binding domain	1b7yB, 39–151	N/A	-
	1b8aA, 1–102	N/A	-
	1bbuA, 64–151	13431467	DNA polymerase II small subunit
		15598836	DNA polymerase III, alpha chain
	1c0aA, 1–106	11261591	DNA polymerase III, alpha chain
		11499379	conserved hypothetical protein
		1169392	DNA polymerase III alpha subunit
		118794	DNA polymerase III alpha subunit
		13620707	putative DNA polymerase III, alpha chain
		14194684	DNA polymerase III alpha subunit
		14194702	DNA polymerase III alpha subunit
		14195653	DNA polymerase III alpha subunit
		14195659	DNA polymerase III alpha subunit
		15594924	DNA polymerase III, subunit alpha
		15598836	DNA polymerase III, alpha chain
		15601899	DnaE
		15642243	DNA polymerase III, alpha subunit
		15669005	M. *jannaschii *predicted coding region MJ0818
		15679404	DNA polymerase delta small subunit
		3914611	ATP-dependent DNA helicase recG
	1cuk, 1–64	N/A	-
	1e1oA, 64–148	11261591	DNA polymerase III, alpha chain XF0204
		14194684	DNA polymerase III alpha subunit
	1fguA, 181–298	15219507	hypothetical protein
		15230563	putative protein
		15790309	Vng1255c from *Halobacterium *sp.
		6166145	DNA polymerase III alpha subunit
		8778702	T1N15.20
	1fl0A	10957481	hypothetical protein
	1g51A, 1–104	14520587	hypothetical protein
		14591565	hypothetical protein
		15595886	hypothetical protein
		3914638	ATP-dependent DNA helicase recG
	1otcB, 36–126	N/A	-
	1quqA, 62–152	15387767	probable replication protein a 28 Kd subunit
	1qvcA, 1–114	N/A	-
Nucleic acid-binding proteins/Cold shock DNA-binding domain like	1a62, 48–125	N/A	-
	1ah9	N/A	-
	1bkb, 75–139	15790688	translation initiation factor eIF-5A; Eif5a
	1c9oA	6014735	Cold shock protein CspSt
	1csp	N/A	-
	1d7qA	N/A	-
	1mjc	N/A	-
	1rl2	N/A	-
	1sro	15671445	N utilization substance protein A
		15794781	N utilisation substance protein A
		15803711	transcription pausing; L factor
	2eifA, 73–132	N/A	-
Nucleic acid-binding proteins/DNA ligase, mRNA capping enzyme, domain2	1a0i, 241–349	N/A	-
	1dgsA, 315–400	N/A	-
	1ckmA, 238–302	N/A	-
	1fviA, 190–293	N/A	-
Nucleic acid-binding proteins/Phage ssDNA-binding proteins	1gpc	N/A	-
	1gvp	N/A	-
	1pfs	N/A	-
Nucleic acid-binding proteins/RNA polymerase subunit RBP8	1a1d	N/A	-
Staphylococcal nuclease/Staphylococcal nuclease	1eyd	13422779	aldose 1-epimerase *
Bacterial enterotoxins/Bacterial AB5 toxins, B units	1c4qA	N/A	-
	1prtF	N/A	-
Bacterial enterotoxins/Superantigen toxins	1an8, 19–94	N/A	-
TIMP-like/Tissue inhibitor of metalloproteases	1ueaB, 14–106	N/A	-
Inorganic pyrophosphatase/ Inorganic pyrophosphatase	2prd	N/A	-
MOP-like/BiMOP, duplicated molybdate-binding domain	1b9mA, 127–262	10639288	probable ATP-binding protein
		10955070	AgtA
		1175513	Putative ferric transport ATP-binding protein afuC
		15598450	probable ATP-binding component of ABC transporter
		3978166	ATPase FbpC
		4895001	glucose ABC transporter ATPase *
Histidine kinase CheA, C-terminal domain/ Histidine kinase CheA, C-terminal domain	1b3qA, 540–671	N/A	-

* Putative false positives as assessed by manual inspection.

### Prediction of functional sites

Ten well-studied protein families (adenylyl kinase, gef, globin, pdz, ph, ptb, ras, sh2, sh3 and subtilase) from the Pfam database (version 7.3) [29] were selected to test the prediction of functional sites. To define functional sites, we considered residues falling within 5Å of any ligand to be functionally important (i.e. AP5 for adenylyl kinase). As a simple quantification of prediction accuracy, we counted the number of predictions that lie within 5Å from the ligands and consider these sites to be true positives.

Our method intends to identify those sites with high similarity within individual sub-trees and high variation among sub-trees. These sites are likely to contribute to functional specificity. Based on a tree partition and the reconstructions at the cutting nodes (details see Methods), we have developed a measure called specificity score (equation (27)). We expect that both highly variable sites and highly conserved sites tend to score low in our method. Ten top-ranking sites were selected as our predicted functional sites for each family. For comparison, we also implemented a simple conservation (SC) method [25], the evolutionary trace (ET) method [26] and the conservation difference (CD) method [21] on the 10 protein families. The results are shown in Table 5. Here, the results from these three methods tend to include invariant or highly conserved sites while the result from our method scores those sites low. Still, the number of true positives of our method is comparable to other methods for several families. For some protein families, such as gef, pdz and subtilase, our method predicts no fewer functional residues than the other three methods.

**Table 5:** Comparison of the true hits among the top 10 predicted sites for ANCESCON, evolutionary trace (ET), simple conservation (SC), and conservation difference (CD) methods

Protein Family	PDB ID^#^	Ligand/ substrate	Number of sites	*	**	***	ANCESCON	ET	SC	CD
adkinase	1aky	AP5	188	42	20	18	3	9.5	9.1	8
gef	1bkd	H-Ras	245	47	4	0	3	3	3	2
globin	1a6g	HEM	147	21	1	1	2	5.5	6	6
pdz	1be9	+	81	15	2	1	6	4	4	2
ph	1mai	I3P	109	11	2	0	2	2	3	2
ptb	1shc	PTR	157	27	2	1	6	5	5	9
ras	821p	GTN	185	29	10	9	2	5.6	8.7	5
sh2	1a09	ACE	83	17	2	1	3	5	4	4
sh3	1nlo	ACE	57	9	1	1	2	5	4	0
subtilase	1av7	SBL	278	22	8	4	5	4.6	3.8	4

^#:^Representative protein structure

*: Number of sites within 5Å to ligand or substrates

**: Number of invariant sites, which may contain gaps

***: Number of invariant sites within 5 Å to ligand or substrates

+: C-terminal peptide of protein CRIPT

**Figure 6:**
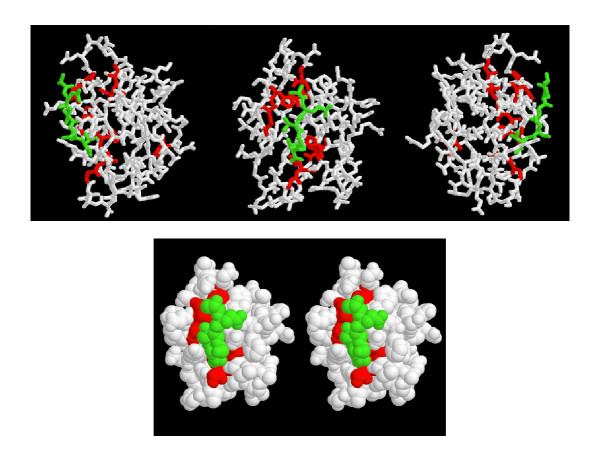
**Mapping top 10 predictions by ANCESCON to PDZ domain (PDB ID: 1be9) [50]. **The color code scheme: ligand is shown in green and the predicted functional residues are shown in red.

Figure 6 shows the mapping of our predictions on the structure for the PDZ domain family. In green color is the ligand and in red color are the functional residues predicted by our method. Six of the predicted residues are within 5Å to the peptide ligand. Nine of the predicted residues are around the ligand binding area. Only one is distant from the ligand (Figure 6).

**Figure 7:**
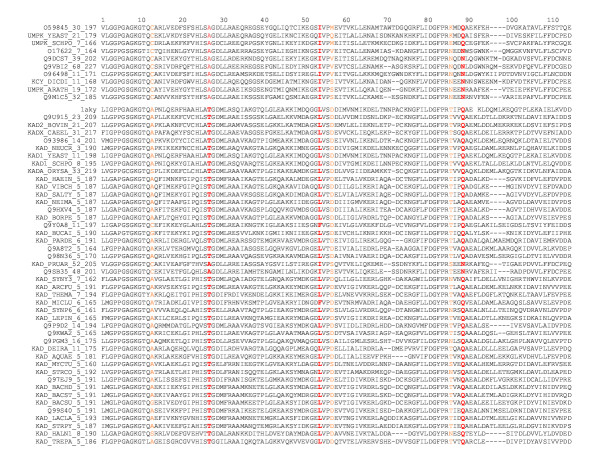
**A partial alignment of the N-terminal part of adenylyl kinases. **Sites colored in red are our predictions that are within 5Å from the ligand. Sites colored in orange are our predictions more than 5Å apart from the ligand.

**Figure 8:**
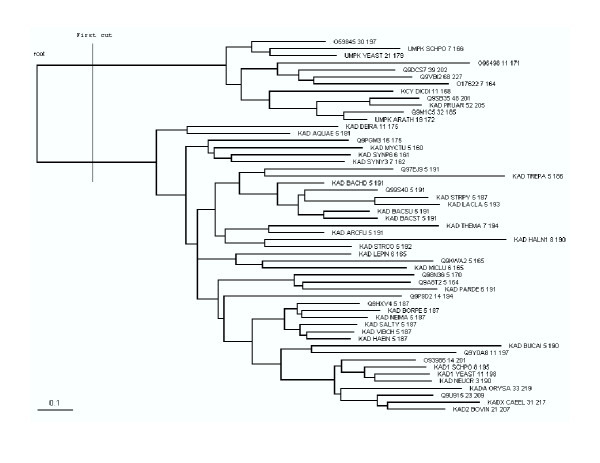
**The evolutionary tree for the adenylyl kinase family generated by "Weighbor". **The first cutting layer is shown. Evolutionary distances are shown to scale.

**Figure 9:**
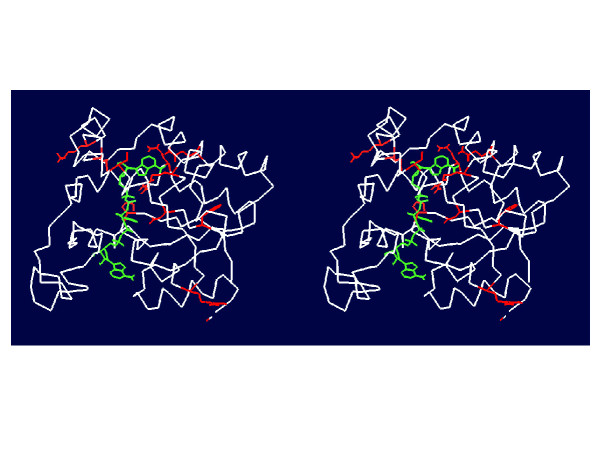
**Mapping top 10 predictions by ANCESCON to adenylyl kinase domain (PDB ID: 1aky) [47]. **The color code scheme: ligand is shown in green and the predicted functional residues are shown in red.

Another example is the family of adenylyl kinases. Our method identified 3 residues within 5 Å to the ligand while the other 3 methods identified more such residues, most of which are in highly conserved positions such as the catalytic residues. Highly conserved residues, however, are not selected by our method since our measure is designed to emphasize on sites contributing to specificity. Figure 7 shows the N-terminal part of the alignment of adenylyl kinases, with our predictions highlighted in red and orange colors. The evolutionary tree for the alignment is shown in Figure 8. The first cutting layer (for details see Methods) results in two well-separated sub-trees. Functional annotations suggest that they contain enzymes with different substrate preferences: adenylyl kinases and uridylate kinases, respectively. Three residues (27, 54 and 89) from our predictions (red colored in Figure 9) contribute to substrate-binding specificity, as have been noted before in the structural studies of the UMP kinases [38]. Figure 9 highlights our predicted functional residues on the adenylyl kinase protein structure. Most of our predictions fall within the specificity pocket.

## Conclusions

We developed a package (ANCESCON) to reconstruct ancestral protein sequences that takes into account the variation of substitution rates among sites. Two methods were proposed to estimate site-specific evolutionary rates (*α*), namely Alignment-Based rate factor (*α*_*AB*_) and rate factor *α *estimated by maximum likelihood (*α*_*ML*_). Consideration of rate variation among sites can alleviate the underestimation of evolutionary distances. Accuracy of ancestral sequence reconstruction by our method is higher than that of PAML, PHYLIP and PAUP* when the given alignment contains more diverse sequences. We show that reconstructed ancestral sequences help to improve detection of distant homologs and prediction of functional sites with specificity.

## Methods

### Transition probability and likelihood calculations

For all models discussed in this paper, we assume all sites in an alignment evolve independently and according to a homogeneous, stationary and time reversible Markov process. The probability of an amino acid *i *to be replaced by amino acid *j *after a time interval *t *is *P*_*ij*_(*t*). The transition probability matrix of 20 amino acids is written as **P**(*t*), which can be calculated as

**P**(*t*) = exp(**Q***t*)     (2)

Here, **Q **is the rate matrix. The non-diagonal elements *q*_*ij *_are the instantaneous rates of change from amino acid *i *to amino acid *j *and diagonal elements *q*_*ii *_are such that each matrix row sums up to 0. **Q **can be calculated by:

**Q **= S* *diag*(*π*)     (3)

**S **is the matrix of amino acid exchangeability parameters [39]. *π*_*i *_is the equilibrium frequency for amino acid *i*. Time reversibility implies that **S **is a symmetric matrix. In our program, the **S **matrix is taken from Whelan and Goldman [39] and the default *π *vector is estimated from the given alignment.

**Q **can be decomposed into eigenvalues (*λ*_*i*_) and eigenvectors (***u***_*i*_).



**U **= (***u***_1_, ..., ***u***_20_)     (5)

**Figure 10:**
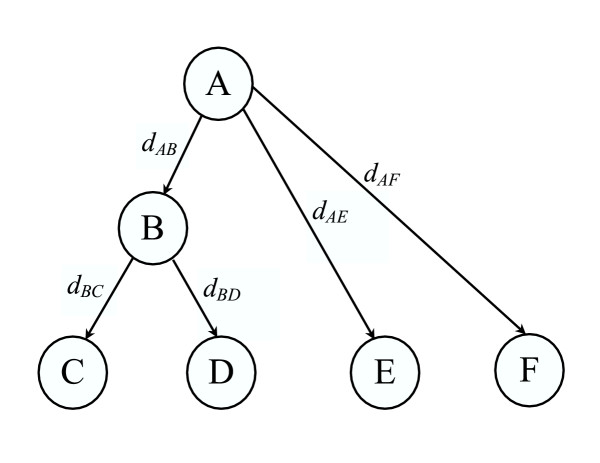
**An evolutionary tree topology. **Nodes C, D, E and F represent given protein sequences, while nodes A and B represent ancestral protein sequences, i.e. unknown sequences. *d*_*YZ *_represents the evolutionary distance between nodes Y and Z.



*P*_*ij*_(*t*) can be calculated using the following equation,



The likelihood function [8] for an evolutionary tree T shown in Figure 10 is:

Here,  is the equilibrium frequency of the amino acid at the node A.  is the transition probability from the amino acid at node A to the amino acid at node B after an evolutionary distance *d*_*AB*_.

Considering that each site *i *has a rate factor *α*_*i *_[13,18], we have:



*t *in equation (6) can be expressed as:

*t *= *α*·*d *    (9)

*d *is the evolutionary distance and *α *is rate factor. The following restriction on the vector *α *holds:



Here, *K *is the number of sites.

### Alignment-Based Rate Factor *α *(*α*_*AB*_) and Rate factor *α *estimated by Maximum Likelihood (*α*_*ML*_)

Our program supports two methods to estimate a rate factor for each site: Alignment-Based rate factor *α *(*α*_*AB*_) and Maximum Likelihood-estimated rate factor *α *(*α*_*ML*_).

The estimation of *α*_*AB *_is empirical and based on the observation that the substitution rate at a site is correlated with the conservation of the site, which, in turn, is correlated with the average transition probability among the amino acids at that site. Conserved sites are dominated by highly similar amino acids and thus have high average transition probabilities among the amino acids. The algorithm to calculate *α*_*AB *_is as follows:

1. Set *t *equal to 1.0 and use equation (6) to calculate a transition probability matrix **P **for 20 amino acids. Equation, , is used to compute a symmetric matrix **P'**.

2. Calculate the average transition probability for each site and take the reciprocal: , where  is the number of non-gapped amino acid pairs in site *i *and the denominator is the sum over the transition probabilities between all amino acid pairs (*j*,*k*) at a site *i*.

3. For invariant sites, *C*_*i *_is set to 0 to make it consistent with the Maximum Likelihood estimation.

4. Equation (11) is used to calculate *α*_*AB*_, so that equation (10) holds.



If an evolutionary tree is assumed for the alignment, we can estimate the *α*_*ML *_factors by maximizing the likelihood (equation (8)) for each site:



If some sites are highly variable, the *α*_*ML *_at those sites can be very large, as has been previously noticed [18]. We consider these rate factors to be outliers. For these sites, we have observed that likelihood changes very little over a wide range of the *α *values. An empirical method is used to reduce the values of *α*_*ML *_outliers, guided by the *α*_*AB *_values. a *Z*-score of the ratio of *α*_*ML *_to *α*_*AB *_is calculated for each site except invariant sites:





Here,  is the ratio of  to  for site *i*;  is the number of sites excluding the invariant sites. If *Z*_*i *_is greater than 3, it is reduced to 3 by decreasing the value of . We repeat this procedure until no *Z*_*i *_for any site *i *is greater than 3. After removing the outliers, we scale the  values so that equation (10) holds.

### Amino acid frequency vector *π *optimization

Two methods are implemented to estimate the equilibrium frequency vector *π*, one derived directly from the given alignment (Alignment-Based *π *or *π*_*AB*_) and the other estimated by Maximum Likelihood (*π*_*ML*_). The likelihood for the entire alignment is a function of *π *with 19 variables. A continuous minimization method by simulated annealing [40] is used to optimize *π*, with the objective function being the logarithm likelihood of the alignment. The simulated annealing is computationally intensive and is the major reason for the long CPU time given in Table 2.

### Distance matrix calculation and tree inference

A Maximum Likelihood approach is used to estimate the evolutionary distances among sequences, either considering rate variation across sites or not. The logarithm likelihood for replacing one protein sequence (A) with another protein sequence (B) after an evolutionary distance *d *can be written as:



Here,  is the equilibrium frequency for the amino acid at site *j *in sequence A.  is the transition probability from amino acid at site *j *in sequence A to amino acid at site *j *in sequence B after an evolutionary distance *α *_*j*_·*d*. *α*_*j *_is 1 if all sites are assumed to evolve at the same rate; otherwise the *α*_*AB *_at site *j *is used for *α*_*j*_.

An estimate of the evolutionary distance between two sequences is obtained by maximizing the likelihood function of equation (15):



Equation (16) can be solved by the bisection root-finding method [40].

After the distance matrix is calculated, the "Weighbor" method, i.e. weighted neighbor joining, is used to infer an evolutionary tree [10].

### Ancestral sequence reconstruction

Two methods are implemented to reconstruct ancestral sequences. One is a marginal reconstruction method [4], and the other is a joint reconstruction method [5]. Below are their brief descriptions.

### The marginal reconstruction method [4]

We calculate *P*(*A*_*r*_|{*A*_*l*_}*T*), which is the conditional probability of amino acid *A*_*r *_at the root, given leaf node amino acid set {*A*_*l*_} and a tree *T*. Since time reversibility is assumed, any internal node can serve as a root. Using Bayes' theorem, we have:



Here, *P*(*A*_*r*_) is used here instead of *P*(*A*_*r*_|*T*) because the frequency of the root amino acid *A*_*r*_, i.e. *π*_*r*_, does not depend on tree *T*. *P*({*A*_*l*_}|*A*_*r*_*T*) is the conditional probability of the known amino acids at the leaf nodes, given *T *and *A*_*r*_. *P*({*A*_*l*_}|*T*) does not depend on *A*_*r*_, so it is calculated as a normalization constant for *P*(*A*_*r*_|{*A*_*l*_},*T*) terms over all 20 possible values of *A*_*r *_to make the sum equal to 1.

For Figure 10, *P*({*A*_*l*_}|*A*_*r*_*T*) can be expanded as:



Here,  is the transition probability from amino acid at node A to amino acid at node B after an evolutionary distance *d*_*AB*_. Equation (18) can be calculated using a recursive method suggested by Felsenstein [8].

If rate factors are used in the reconstruction of the root sequence, we have:



Here, *α*_*i *_could be either *α*_*AB *_or *α*_*ML *_at site *i*. *P*(*A*_*C*_,*A*_*D*_,*A*_*E*_,*A*_*F *_| *A*_*A*_,*T*)_*i *_is the conditional probability *P*(*A*_*C*_,*A*_*D*_,*A*_*E*_,*A*_*F *_| *A*_*A*_,*T*) at site *i*.

### The joint reconstruction method [5]

The objective of a joint reconstruction method is to find the combination of amino acids for an internal node set {*A*_*i*_} that maximize the conditional probability of this amino acid combination, given the leaf node amino acid set {*A*_*l*_} and a tree *T*, *P*({*A*_*i*_}|{*A*_*l*_},*T*). Using the Bayes' theorem, we have:



Because *P*({*A*_*l*_}|*T*) is the same for all amino acid combination at internal node set {*A*_*i*_} this problem becomes finding the maximum of *P*({*A*_*l*_}|{*A*_*i*_},*T*) *P({*A*_*i*_}).

The details of a fast algorithm to solve equation (20) can be found in Pupko et al. [5]. We also incorporated site-specific rate factors in this algorithm, in a similar way as equation (19)

### Gaps

Due to difficulties with the probabilistic models of gaps, a simplified empirical approach is used to alleviate the problem. We assume that gaps are "supersede" letters. Gaps are considered for each site independently. If a leaf node has a gap instead of an amino acid at a site, this node will be deleted from the tree for this site. After dealing with leaves, we check all internal nodes for children. If an internal node has no children or only one child due to the leaf removal because of gaps, it will be removed from the tree and a gap will be assumed as its reconstructed state.

### Simulations of evolutionary process

Two methods of simulating amino acid substitution process were used to test the reliability of reconstruction, rate factors and evolutionary distance estimation. The first simulation method was based on a homogeneous time reversible Markov model. The parameters from Whelan and Goldman [39] were chosen for our model, including the equilibrium frequency vector *π *and the **S **matrix. Given the length of a branch from a parent node to one of its child nodes and the amino acid for the parent node, we simulated the substitution process to generate an amino acid for the child node based on the transition probabilities that were calculated using equation (6). For the arbitrarily selected tree shown in Figure 2, we first generated a random sequence of 100 amino acids as the root sequence based on the amino acid frequencies from Whelan and Goldman [39]. We then simulated the random substitution process to obtain all leaf node sequences. This simulation was repeated 100 times. The resulting 100 alignments were used to test the reliability of the reconstruction result. In this simulation, each site evolved independently according to the same tree topology and branch lengths, thus there was no rate heterogeneity across sites.

The second simulation method, based on a *Z*-score model, introduced rate variation across sites by using structural and functional information for a specific protein family [21]. We selected three protein families for the *Z*-score simulations under structural and functional constraints: pdz domain (Protein DataBank (PDB) ID: 1g9o) [41], trypsin (PDB ID: 1sgt) [42] and carboxypeptidase A (PDB ID: 2ctb) [43]. Given a rooted tree, the native sequence with known structure was used as the root sequence. Simulations were made along the tree to generate sequences at any internal node or leaf node. If the evolutionary distance from a parent node to a child node was *d*, the child sequence was obtained after l*d accepted substitutions starting from the parent sequence, where *l *is protein sequence length. Simulations of the substitution process were repeated 100 times. For each site, the number of accepted substitutions was recorded and averaged over 100 simulations. Rate factors (observed *α*), representing site mutability, were calculated from these average substitution numbers, such that the average of rate factors is 1 (equation (10)). 100 simulated alignments were used to test the rate factor estimators (*α*_*AB *_and *α*_*ML*_), distance calculation methods and ancestral sequence reconstruction.

### Homology detection

#### Testing dataset

38 OB (Oligonucleotide/oligosaccharide binding)-fold [34] proteins with known structures were selected for homology detection test. OB-fold has a 5-stranded *β*-barrel structure. In the SCOP (Structure Classification of Proteins) database (version 1.55) [44], there are 7 OB-fold superfamilies. The superfamily of nucleic acid binding proteins is the most populated. Diversity of many OB-fold homologs extends beyond detection by automatic PSI-BLAST searches. Multiple sequence alignments of native sequences were obtained from PSI-BLAST searches starting from the 38 OB-fold sequences with known structures. We also selected 10 alignments (adenylyl kinase, gef, globin, pdz, ph, ptb, ras, sh2, sh3 and subtilase) from the Pfam database (version 7.3) [29] for homology detection test.

#### Four different methods

For each alignment with *N *sequences, ancestral sequences for the *N*-1 internal nodes were reconstructed. The idea is to test whether adding more sequences to a native alignment can help homology detection. Four types of combined alignments were generated, adding different sets of *N*-1 sequences to the native alignment. In the first case, the added sequence at each internal node consisted of amino acids with the largest probability at each position. In the second case, the added sequences were made up of amino acids with the second largest probability. In the third case, we shuffled the native alignment at each position while keeping the gap pattern as in the native alignment. After shuffling, we added *N*-1 sequences resulted from the shuffling to the native alignment. In the fourth case, *N*-1 random sequences were generated with the overall amino acid frequencies of the native alignment. These four methods are named "BEST", "SECOND BEST", "SHUFFLE" and "RANDOM", respectively.

### Prediction of functional sites

Our objective is to find sites that are well conserved within each sub-tree, but show high variability between different sub-trees. These sites are likely to contribute to functional specificity [26,45,46].

#### Sequence datasets

Multiple sequence alignments of ten protein families were chosen from the Pfam database (version 7.3) [29]. These families are: adenylyl kinase (adkinase) (representing structure PDB ID: 1aky; its ligand or substrate: AP5) [47], guanine nucleotide exchange factor (gef) (1bkd; H-Ras) [48], globin (1a6g; HEM) [49], pdz domain (1be9; C-terminal peptide of protein CRIPT) [50], ph domain (1mai; I3P) [51], ptb domain (1shc; PTR) [52], ras (821p; GTN) [53], sh2 domain (1a09; ACE) [54], sh3 domain (1nlo; ACE) [55] and subtilase (1av7; SBL) [56]. Most of these alignments contain many sequences. We pruned and clustered the sequences in each alignment according to the length and diversity. Representative sequences were kept and used for tree inference and ancestral sequence reconstruction. This procedure was done in three steps: 1) removing fragments, 2) single-linkage clustering and 3) complete-linkage clustering, as described below.

1. For each family, there is a template sequence with known structure. The sequences, which cover less than 75% of the non-gapped positions in the template sequence with amino acids, were considered to be fragments and discarded.

2. A sequence identity matrix was calculated for the remaining sequences. A single linkage clustering was done to form sequence groups at sequence identity threshold 0.8. For each group, we chose the longest sequence as a representative, discarding other members. This step reduced redundancy in the dataset.

3. An average sequence identity was calculated for the remaining sequences. We used this average identity as a threshold for complete linkage clustering to form new sequence groups. Four groups with the largest sequence numbers were chosen to form our new alignment. Any group with the same number of sequences as the fourth group was also included in the new alignment. The purpose of this step is to keep the major sequence subgroups of a family while leaving out highly divergent sequences that might be deleterious for tree inference.

#### Rooting

The "Weighbor" method gives an unrooted tree. For our purpose of predicting functional sites, we need to find a point on the tree that serves as the root. We used a least-squares modification of the midpoint rooting procedure to define the root [57].

#### Tree partitioning

**Figure 11:**
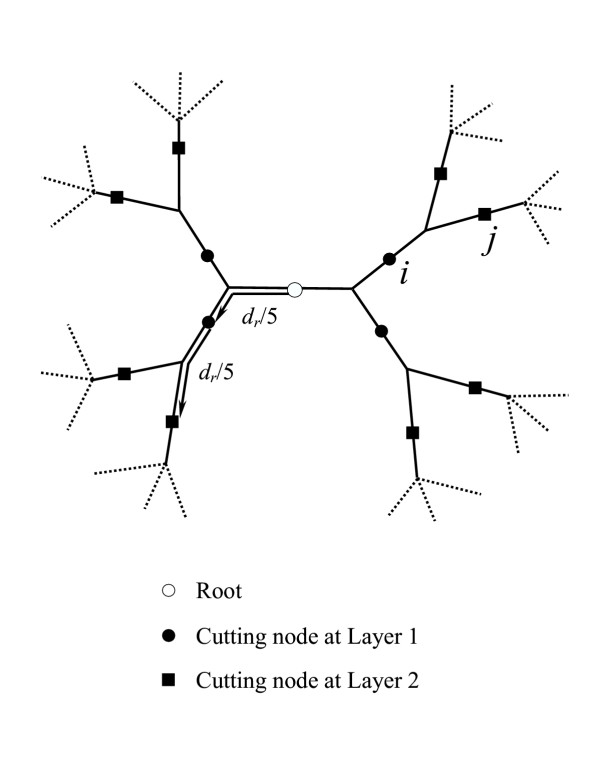
**An example showing the different cutting layers in a rooted tree. ***d*_*r *_is the average distance from the root to all leaf nodes. Nodes *i *and *j *are neighboring cutting nodes.

The tree was partitioned into sub-trees at several levels and compared the amino acid usages within each sub-tree and among the sub-trees. For this partitioning, we "cut" the tree into a fixed number of equal-distanced layers, using the midpoint as the root (Figure 11). Several criteria were tried for selecting the distance between adjacent layers. Empirically we found that a simple partition of the tree into 5 layers usually gave the best results. If the average distance from the root to all leaf nodes is *d*_*r*_, then the distance between adjacent layers is *d*_*r*_/5 (Figure 11). Each place of a "cut" between the layers corresponds to a certain ancestral sequence. We term the location of a "cut" as a "cutting" node. The marginal reconstruction method was used to reconstruct amino acid probability vectors for all the cutting nodes (Figure 11). The reconstructed probability vector of a cutting node reflects the amino acid usages of the sub-tree under it.

#### Calculating specificity score for each site

We use {*L*_*K*_} to represent the set of cutting nodes for layer *L*_*K*_, *K *= 0,1,5. {*L*_*0*_} is the root and *L*_1 _is the closest layer to the root, etc.

A dissimilarity score between any neighboring cutting node pair is calculated. The definition of a neighboring cutting node pair (*i*, *j*) (Figure 11) is:

1. *i *∈ {*L*_*K*_}

2. *j *∈ {*L*_*K*+1_}

3. Node *i *is an ancestor of node *j *(all points on the path from *j *to root node are ancestors of node *j*), so that the distance between *i *and *j *is exactly *d*_*r*_*/5*. Each cutting node has only one ancestral cutting node neighbor.

The dissimilarity score for cutting node *j *and its ancestral cutting node neighbor *i, *i.e. *anc*(*j*), at site *m *is defined as:



 and  are the reconstructed probabilities of amino acid *A *at cutting node *j *and its ancestral cutting node neighbor *i*(*anc*(*j*)), respectively.

Let , *K *= 1,...,5     (22)

Here,  is the average dissimilarity score for layer *K *. *N*_*K *_is the number of cutting nodes in layer *K*.

The specificity score is defined as:



 reflects the difference of amino acid compositions among the major sub-trees defined by the first layer.  to  reflect the average difference of amino acid compositions within each sub-tree. If the amino acids are highly conserved within each sub-tree but show variability among the sub-trees,  to  are small and  is large, leading to a large value of *S*_*m*_. We set *S*_*m *_to 0 for invariant sites. We sort the sites by their specificity scores and choose the 10 top scoring sites as our predicted functional sites. Those predicted functional sites that lie within 5 Å from the ligand(s) are considered to be true positives.

#### Comparison with other methods

We compared our method with three other methods for prediction of functional sites. The first method (Simple Conservation or SC) is based on sequence conservation. Highly conserved sites are considered to be functional. For each family, we sorted the sites by positional conservation [25] and chose the 10 top-ranking sites as the predictions. There might be ties for sites. For example, if there were 5 sites tied at the tenth conservation value and only one of them was within 5Å from the ligand(s), then its contribution to the total number of "correct predictions" was 1/5. The second method is the evolutionary trace (ET) method [26], which partitions a sequence identity dendrogram into sub-trees at varying sequence identity thresholds. Sites that are invariant within each individual sub-tree are picked as functional sites. A higher identity threshold gives rise to more sub-trees and, since conserved sites are more frequent in the sub-trees with smaller sizes, lead to more predicted sites. ET analysis was performed from a low identity threshold to higher thresholds until the number of predicted sites was 10 or just above 10 (in the cases of ties). Ties were resolved similarly to the simple conservation method. The third method (conservation difference or CD) is based on the conservation differences between a native alignment and an alignment derived from the *Z*-score sequence design [21]. The basic idea was to differentiate sites conserved due to structural stability and sites conserved due to function. Since the pairwise potential in the *Z*-score design tends to weaken the conservation caused by function, functionally conserved sites tend to have a large conservation difference between the native alignment and the alignment of designed sequences. We chose 10 top ranking sites sorted by conservation difference as predictions by CD.

## Authors' contributions

NVG conceived and initiated the study. All authors took part in developing methods and designing experiments. WC wrote the source code and JP analyzed the data. All authors read and approved the final manuscripts.
